# Improvement of Morphine-Mediated Analgesia by Inhibition of β-Arrestin 2 Expression in Mice Periaqueductal Gray Matter

**DOI:** 10.3390/ijms10030954

**Published:** 2009-03-05

**Authors:** Yuting Li, Xing Liu, Chang Liu, Jiuhong Kang, Jingyu Yang, Gang Pei, Chunfu Wu

**Affiliations:** 1 Department of Pharmacology, Shenyang Pharmaceutical University, Shenyang 110016, P.R. China; E-Mails: genggeng800816@yahoo.com (Y.L.); yangjingyu2006@gmail.com (J.Y.); gpei@sibs.ac.cn (G.P.); 2 Laboratory of Molecular Cell Biology, Institute of Biochemistry and Cell Biology, Shanghai Institutes for Biological Sciences, Chinese Academy of Sciences, Shanghai 200031, P.R. China; E-Mails: lonelyrunner27@yahoo.com.cn (C.L.); jhkang@sibs.ac.cn (J.K.); 3 The State Key Laboratory of Medical Neurobiology, Shanghai Medical College and Institutes of Brain Science, Fudan University, Shanghai 200032, P.R. China; E-Mail: xingliu@fudan.edu.cn

**Keywords:** β-Arrestin 2, Lentivirus, Periaqueductal gray, Analgesia, Morphine

## Abstract

Morphine is a well-known μ-opioid receptor (MOR) agonist and an efficient analgesic, but its long-term use inevitably leads to drug addiction and tolerance. Here, we show that specific inhibition of β-arrestin2 with its siRNA lentivirus microinjected in mice periaqueductal gray matter (PAG) significantly improved both acute and chronic morphine analgesia and delayed the tolerance in the hotplate test. The specific effect of β-arrestin2 was proven by overexpression or knockdown of its homology β-arrestin1 in PAG, which showed no significant effects on morphine analgesia. These findings suggest that specific siRNA targeting β-arrestin2 may constitute a new approach to morphine therapy and other MOR agonist-mediated analgesia and tolerance.

## 1. Introduction

Morphine has long been used as a clinical pain-relieving agent. However, the antinociceptive efficacy of morphine decreases over prolonged courses of treatment and long-term application of morphine will inevitably produce tolerance. Moreover, long-term use of morphine also leads to addiction. A large body of evidence has shown that stimulation of μ-opioid receptor (MOR) is not only one of the main mechanisms for morphine analgesia, but also the main mechanism for morphine-induced tolerance and addiction [1–3].

β-Arrestins, including β-arrestin 1 and β-arrestin 2, are highly expressed in the central nervous system (CNS) [4] and their critical role in regulating MOR in the CNS has been demonstrated. In β-arrestin 2 knockout mice, the antinociceptive effects of morphine are remarkably enhanced and prolonged, while the tolerance to the antinociceptive effects is significantly attenuated in both hot-plate and tail-flick tests [5,6] with the mechanisms of desensitization of the MOR [7,8]. Coincidently, as MOR agonists produce antinociceptive tolerance, the expression of β-arrestin 2 is significantly increased in locus coeruleus, cortex and striatum [9–11], while the intrathecal administration of β-arrestin 2 antisenses delays the development of tolerance to morphine [12]. Thus, regulating the expression of β-arrestin 2 in special regions of CNS may be one approach to improve the antinociceptive effect of morphine and delay its tolerance.

Morphine is known to produce analgesia by activating MOR via supraspinal and spinal-related pathways finally located to PAG. PAG is thus widely considered to be a main output pathway of the limbic system and in turn, control pain transmission [13]. Moreover, it has been reported that the antinociceptive effects of morphine is attenuated in rats receiving microinjections of β-arrestin 2 adenovirus in PAG [14]. In the present study, we investigated the effects of β-arrestin 2 in mice PAG by locally and transiently delivering β-arrestin 2 or its specific siRNA lentiviruses on acute and chronic morphine-induced analgesia and tolerance.

## 2. Results and Discussion

### 2.1. Expression of β-arrestins mediated by lentiviruses in mouse PAG

**Figure 1.**
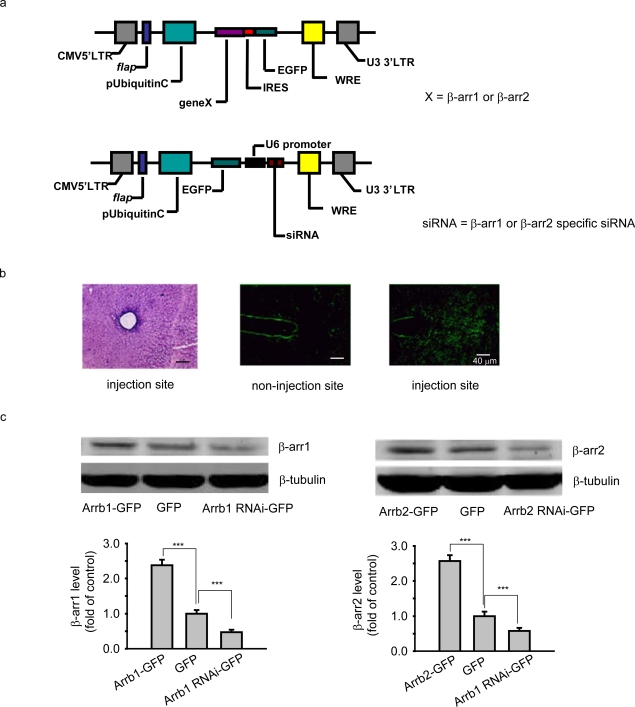
(a) Map of β-arrestin recombinant lentivirus generated by FUGW vector. (b) HE stain (left) and confocal images of the non-injection (middle) and injection site (right) in the PAG taken from an animal microinjected with the β-arrestin 2 lentivirus. And similar images of other lentiviruses used in the study were observed (data not shown). Green, GFP. Scale bar: 40 μm. (c) Immunoblot analysis of β-arrestin protein levels in the microinjection sites. β-arr1, β-arrestin 1; β-arr2, β-arrestin 2. n = 3–4 mice of each group. ***p < 0.001 versus the GFP lentivirus.

We first constructed the lentiviruses carrying human β-arrestin 1 and β-arrestin 2 or specific siRNA targeting mouse β-arrestin 1 or β-arrestin 2 (Figure 1a), and performed histochemical experiment and Western Blot analysis to determine whether microinjection of these lentiviruses in mouse PAG could mediate the ectopic expression of β-arrestin 1 and β-arrestin 2. As shown in Figure 1b, GFP fluorescence was detected in PAG of the injection site but not in the non-injection sites, and no injury was observed at the injection site using HE staining. And microinjection of GFP tagged β-arrestin 1 (Arrb1-GFP) or β-arrestin 2 (Arrb2-GFP) lentivirus in PAG significantly upregulated the protein levels of β-arrestin 1 and β-arrestin 2 while microinjection of GFP tagged β-arrestin 1-spcific siRNA (Arrb1-siRNA-GFP) or β-arrestin 2-specific siRNA (Arrb2-siRNA-GFP) clearly downregulated their protein levels, respectively (Figure 1c).

### 2.2. Enhancement of acute and chronic morphine analgesia in mice microinjected with β-arrestin 2 specific siRNA lentivirus in PAG

**Figure 2.**
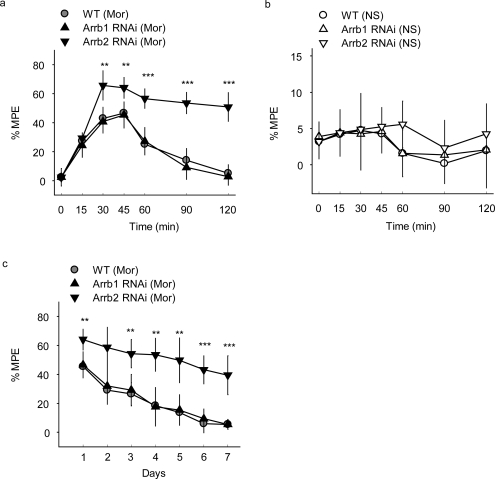
(a and b) Acute morphine analgesia in mice microinjected in PAG with GFP tagged β-arrestin 1-specific siRNA (Arrb1-RNAi), β-arrestin 2-specific siRNA (Arrb2-RNAi) or GFP control (WT) lentivirus. Mor, morphine; NS, normal saline, n = 11–12 mice of each group. Two-way ANOVA, **p < 0.01, ***p < 0.001 versus the WT lentivirus mice administrated with morphine. **(c)** Chronic morphine analgesia in mice microinjected in PAG with Arrb1-RNAi, Arrb2-RNAi or WT lentivirus, n = 9–10 mice of each group. Two-way ANOVA, **p<0.01, ***p < 0.001 versus the WT lentivirus mice administrated with morphine.

The morphine-induced analgesia test was then carried out in mice microinjected with β-arrestin-specific siRNA lentivirus and the analgesia was evaluated by measuring response latencies in the hot-plate test. A dose of 10 mg/kg morphine and a route of subcutaneous administration that are known to induce analgesia in mice were applied. After a single administration of 10 mg/kg morphine subcutaneously (s.c.), the mice injected with Arrb2-siRNA-GFP lentivirus in PAG showed an significantly enhanced and prolonged antinociceptive response in the hotplate test compared with the control mice injected with WT-GFP lentivirus, with the MPE of 46% ± 7% in WT-GFP group and 64% ± 7% in Arrb2 siRNA-GFP group at 30–45 min after morphine treatment (Figure 2a).

In contrast, mice exposed to normal saline (NS) showed no significant differences between groups with GFP, Arrb1-siRNA-GFP or Arrb2-siRNA-GFP mice (Figure 2b), indicating that microinjection of β-arrestin 2 specific siRNA could only influence morphine specifically induced analgesia in this study. The mice microinjected with β-arrestin-specific siRNA lentivirus in PAG were also exposed to chronic morphine. Similar with the acute morphine treatment, the hot-plate latencies were significantly enhanced in Arrb2-siRNA-GFP mice for 7 days (Figure 2c) and this significance could last for 15 days (data not shown). However, knockdown of its homology β-arrestin 1 in mice PAG by infection with Arrb1-siRNA-GFP lentivirus did not show any significant differences in both acute and chronic morphine-induced analgesia as compared with the control mice (Figures 2a and 2c). Thus, these results reveal that β-arrestin 2 plays critical role in morphine analgesia in PAG differently from the roles of β-arrestin 1 in regulation of MOR-mediated signal transduction and opioid functions in mice PAG.

### 2.3. Attenuation of acute and chronic morphine analgesia in mice microinjected with β-arrestin 2 lentivirus in PAG

**Figure 3.**
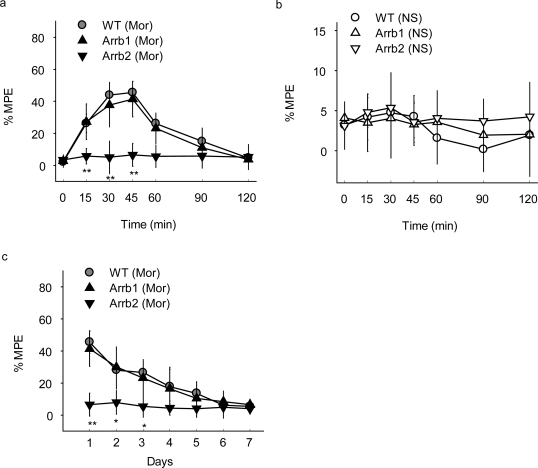
(a and b) Acute morphine analgesia in mice receiving PAG microinjection of GFP tagged β-arrestin 1 (Arrb1), β-arrestin 2 (Arrb2) or GFP control (WT) lentiviruses. Mor, morphine; NS, normal saline, n = 10–12 mice of each group. Two-way ANOVA, **p < 0.01 versus the WT lentivirus mice administrated with morphine. (c) Chronic morphine-induced analgesia in mice receiving PAG microinjection of Arrb1, Arrb2 or WT lentiviruses. n = 9–10 mice of each group. Two-way ANOVA, **p < 0.01, *p < 0.05 versus the WT lentivirus mice administrated with morphine.

The effect of β-arrestin 2 on morphine analgesia was also investigated in mice microinjected in PAG with lentiviruses carrying β-arrestin 1 or β-arrestin 2. In acute and chronic morphine treatment, the mice overexpressed β-arrestin 2 in PAG by infection with Arrb2-GFP lentivirus experienced a significantly shortened and attenuated antinociceptive response in the hotplate test compared with the control mice microinjected with GFP lentivirus (Figures 3a and 3c). To the contrary, microinjection of β-arrestin 1 lentivirus in mice PAG showed no significant changes in either acute or chronic morphine-induced analgesia (Figures 3a and 3c). Similar with the microinjection of siRNA lentivirus, mice exposed to normal saline (NS) showed no significant differences between groups with GFP, Arrb1-GFP and Arrb2-GFP mice (Figure 3b). Thus, these results provide further proof on the enhanced analgesic effects of morphine by knockdown of β-arrestin 2.

Morphine is a classic opiate analgesic in pain treatment in conditions ranging from acute pain, postoperative pain to chronic pain including cancer pain [15,16]. These clinical uses of morphine are often required for extended periods. However, prolonged administration of morphine in chronic pain states often leads to antinociceptive tolerance. For obtaining the same analgesia effect, higher doses of the drug have to be used, resulting in serious side effects such as constipation, mental alertness impairment, etc. In the present study, we discovered that knockdown of β-arrestin 2 locally in PAG could enhance morphine analgesia and ameliorate the development of tolerance.

It is known that morphine produces analgesic effects mainly through stimulation of MOR [1–3], which is desensitized by β-arrestins. β-Arrestins include β-arrestin 1 and β-arrestin 2, both of which are of high homology [17] and high expression in the CNS [4], but potential functional differences between β-arrestin 1 and β-arrestin 2 have been observed [18–20]. In the present study, we found that β-arrestin 1 and β-arrestin 2 differentially regulate acute and chronic morphine-induced antinociceptive responses. Interfering in the expression of β-arrestin 2, but not β-arrestin 1 in PAG could effectively mediate MOR signaling in morphine-induced analgesia. The difference may be due to the following reasons: 1) β-Arrestin 2 appears to be more abundant than β-arrestin 1 in CNS [4]. 2) Although both β-Arrestin 1 and β-arrestin 2 function as important molecules in the agonist-stimulated desensitization of MOR [21,22], β-arrestin 2 is more efficient than β-arrestin 1 in translocating to the membrane upon stimulation of MOR [20]. Correspondingly, it has been found that acute and chronic morphine treatments differentially alter β-arrestin 1 and β-arrestin 2 mRNA levels in cerebral cortex, PAG, and locus coeruleus in rats. No significant change of β-arrestin 1 mRNA level was observed in either PAG or cerebral cortex following the acute or chronic morphine administration, while β-arrestin 2 mRNA level in these regions showed significant changes in response to morphine treatment [23], indicating the probable different roles of β-arrestin 1 and β-arrestin 2 in response to MOR signaling and the potential therapeutic application of β-arrestin 2 knockdown in the morphine antinociceptive tolerance.

β-Arrestins have been found to be ubiquitously expressed in different cells and tissues [4]. Considering the important role of β-arrestins in GPCR signaling [24,25], knockdown of β-arrestin 2 in a specific analgesia-related region may be more suitable for clinic therapy. Supraspinal regions that are shown to express opioid receptors and correlate to antinociception include nucleus accumbens, frontal cortex, hippocampus, PAG, thalamus and hypothalamus [26,27]. PAG is extensively accepted as the prominent region in opioid-mediated antinociception and is found to be rich in MOR [27] Therefore, PAG is an ideal region for gene targeting in pain relieving therapy. Our results showed that knockdown of P-arrestin 2 in PAG could greatly enhance morphine-induced analgesia and delayed the production of tolerance, suggesting the application of the lentivirus carrying siRNA specifically targeting P-arrestin 2 in PAG is a potent therapeutic strategy for morphine and other MOR agonists-induced antinociceptive tolerance.

## 3. Experimental Section

Mice used in this study were 3 month-old males weighing between 26 and 30 g. Experiments were conducted in accordance with National Institutes of Health Guide for the Care and Use of Laboratory Animals. Morphine sulfate was purchased from Shenyang Pharmaceutical Company, PR. China. The compounds were injected at a volume about 100 μL/g animal weight.

Human β-arrestin 1 or β-arrestin 2 was first constructed into the vector of pIRES2-EGFP and further subcloned into FUGW backbone vector. Mouse β-arrestin 1 or β-arrestin 2 siRNA sequence from pBSU6 vector was subcloned into FUGW backbone vector. Then the FUGW plasmid encoding GFP, GFP tagged human β-arrestin 1 (Arrb1-GFP) or β-arrestin 2 (Arrb2-GFP), or GFP tagged β-arrestin 1-specific siRNA (Arrb1-siRNA-GFP) or β-arrestin 2-specific siRNA (Arrb2-siRNA-GFP, 5’ACGTTGACATTAAGGGGCTCCC3’) was co-transfected with packaging vectors (VsVg and PAX2) into HEK-293T cells to generate recombinant lentivirus. Viruses were harvested and collected to give the titers about 1.0×10^7^ CFU/mL. For surgery and microinjection, chloral hydrate-anesthetized mice were positioned in a stereotaxic apparatus (NARISHIGE Scientific Instrument LAB); the lentivirus filled in the glass electrode was injected aiming at PAG (AP-4.5 mm; ML ±0 mm; DV 2mm). Lentivirus (1.5 μL) was microinjected into the PAG at a rate of 0.15 μL/min. The glass electrode was left in place for additional 2 min. The expression of the aim protein was detected and the behavioral experiment was conducted 10-13 days after virus microinjection.

For Western Blot analysis, the brain was acutely dissected and PAG was separated in 5 minutes on ice. Then the section was homogenized with buffer including 50 mM Tris, 250 mM sucrose, 25 mM KCl and 0.5 mM PMSF. The lysates were centrifuged at 5500 × g for 10 min at 4 °C. The supernatants were collected and protein concentrations of the samples were determined using Micro BCA Protein Assay Kit (Pierce Biotechnology, Rockford, USA). Forty micrograms of protein from each sample were loaded on a 10% SDS-polyacrylamide gel and transferred to a nitrocellulose membrane. The membrane was then incubated with mouse anti-β-arrestin 1 antibody (BD Biosciences, San Jose, CA) and anti-β-arrestin 2 (Santa Cruz) and further incubated with secondary antibodies conjugated to IRDye700 (1:5,000) or IRDye800 (1:5,000). Blotted proteins were detected using the Odyssey infrared imaging system (Li-COR Biosciences, Lincoln, Nebraska).

For histochemical experiment, the animals were anesthetized and perfused with NS, followed by iced 4% paraformaldehyde. Then the tissues were post-fixed with 4% paraformaldehyde at 4 °C for 2-4 hrs and stored in 20% PBS-buffered sucrose solution for 36 hrs. Then frozen coronal brain sections (14 um) were cut on a freezing microtome and washed by 1 × PBS for three times with each time of 5 min. Then, the slices were analyzed under the fluorescence microscope (Zeiss Axio Observer Z1) or performed Haematoxylin-Eosin (HE) Staining.

Hot-plate experiments were performed as described previously [5]. In each antinociception assay, nociceptive latencies were assessed as the response time to the hot plate. The ‘response’ was defined by the animal either licking its fore or hind paws or flicking its hind paws. In the studies, the most prominent response was forepaw licking and hind paw flicking. When exposed to the test under morphine treatment, the time limit is 30 sec to the thermal source, thus preventing prolonged painful stimulation and tissue damage. Such data recorded in each mouse are analyzed and presented as the percentage of maximum possible effect (%MPE), which is calculated by the following formula: 100% × [(drug response time-basal response time)/(30 sec-basal response time)] = %MPE. For acute antinociceptive tolerance test, antinociceptive responses were measured as hot-plate response latency at 52 °C after a single morphine treatment (10 mg/kg, sc). For chronic morphine treatment, mice were treated daily (between 13:00 and 14:00) with morphine (10 mg/kg, s.c.) and antinociception was assessed 30 min later on 52 °C hotplate for every day for 7 days.

The quantitative data are represented as mean ± s.e.m., and analyzed using Sigma Stat 3.5. Two-way analysis of variance was used to compare differences in hot-plate latency between groups.

## 4. Conclusions

Specific inhibition of β-arrestin 2 with its siRNA lentivirus microinjected in mice PAG significantly improved both acute and chronic morphine analgesia and delayed the tolerance. On the contrary, overexpression of β-arrestin 2 in PAG of mice with its lentivirus significantly attenuated the analgesic effects of acute and chronic morphine. Overexpression or knockdown of β-arrestin 2 homology β-arrestin 1 in PAG showed no significant effects on morphine analgesia. The present findings suggest a new approach of specific siRNA targeting β-arrestin 2 to the therapy of morphine and other MOR agonists-mediated analgesia and tolerance.
